# Newly formed dust within the circumstellar environment of SN Ia-CSM 2018evt

**DOI:** 10.1038/s41550-024-02197-9

**Published:** 2024-02-09

**Authors:** Lingzhi 灵芝 Wang王, Maokai Hu, Lifan Wang, Yi 轶 Yang 杨, Jiawen Yang, Haley Gomez, Sijie Chen, Lei Hu, Ting-Wan Chen, Jun Mo, Xiaofeng Wang, Dietrich Baade, Peter Hoeflich, J. Craig Wheeler, Giuliano Pignata, Jamison Burke, Daichi Hiramatsu, D. Andrew Howell, Curtis McCully, Craig Pellegrino, Lluís Galbany, Eric Y. Hsiao, David J. Sand, Jujia Zhang, Syed A. Uddin, J. P. Anderson, Chris Ashall, Cheng Cheng, Mariusz Gromadzki, Cosimo Inserra, Han Lin, N. Morrell, Antonia Morales-Garoffolo, T. E. Müller-Bravo, Matt Nicholl, Estefania Padilla Gonzalez, M. M. Phillips, J. Pineda-García, Hanna Sai, Mathew Smith, M. Shahbandeh, Shubham Srivastav, M. D. Stritzinger, Sheng Yang, D. R. Young, Lixin Yu, Xinghan Zhang

**Affiliations:** 1 Chinese Academy of Sciences South America Center for Astronomy (CASSACA), National Astronomical Observatories, CAS, Beijing, China; 2 CAS Key Laboratory of Optical Astronomy, National Astronomical Observatories, Chinese Academy of Sciences, Beijing, China; 3 Purple Mountain Observatory, Chinese Academy of Sciences, Nanjing, China; 4 George P. and Cynthia Woods Mitchell Institute for Fundamental Physics and Astronomy, Texas A&M University, Department of Physics and Astronomy, College Station, TX USA; 5 Physics Department and Tsinghua Center for Astrophysics (THCA), Tsinghua University, Beijing, China; 6 Department of Astronomy, University of California, Berkeley, CA USA; 7 Cardiff Hub for Astrophysics Research and Technology, School of Physics & Astronomy, Cardiff University, Cardiff, UK; 8 McWilliams Center for Cosmology, Department of Physics, Carnegie Mellon University, Pittsburgh, PA USA; 9 Graduate Institute of Astronomy, National Central University, Jhongli, Taiwan; 10 Beijing Planetarium, Beijing Academy of Science and Technology, Beijing, China; 11 European Organisation for Astronomical Research in the Southern Hemisphere (ESO), Garching b. München, Germany; 12 Department of Physics, Florida State University, Tallahassee, FL USA; 13 Department of Astronomy, University of Texas, Austin, TX USA; 14 Instituto de Alta Investigación, Universidad de Tarapacá, Arica, Chile; 15 Millennium Institute of Astrophysics (MAS), Santiago, Chile; 16 Las Cumbres Observatory, Goleta, CA USA; 17 Department of Physics, University of California, Santa Barbara, CA USA; 18 Center for Astrophysics, Harvard & Smithsonian, Cambridge, MA USA; 19 The NSF AI Institute for Artificial Intelligence and Fundamental Interactions, Alexandria, VA USA; 20 Institute of Space Sciences (ICE, CSIC), Barcelona, Spain; 21 Institut d’Estudis Espacials de Catalunya (IEEC), Barcelona, Spain; 22 Department of Astronomy and Steward Observatory, University of Arizona, Tucson, AZ USA; 23 Yunnan Observatories, Chinese Academy of Sciences, Kunming, China; 24 European Southern Observatory, Santiago, Chile; 25 Department of Physics, Virginia Tech, Blacksburg, VA USA; 26 Astronomical Observatory, University of Warsaw, Warszawa, Poland; 27 Carnegie Observatories, Las Campanas Observatory, La Serena, Chile; 28 Department of Applied Physics, School of Engineering, University of Cádiz, Cádiz, Spain; 29 Astrophysics Research Centre, School of Mathematics and Physics, Queen’s University Belfast, Belfast, UK; 30 Departamento de Ciencias Físicas, Universidad Andres Bello, Santiago, Chile; 31 Université de Lyon, Université Claude Bernard Lyon 1, Villeurbanne, France; 32 Space Telescope Science Institute, Baltimore, MD USA; 33 Department of Physics and Astronomy, Aarhus University, Aarhus, Denmark; 34 Henan Academy of Sciences, Zhengzhou, China

**Keywords:** Time-domain astronomy, Astrophysical dust

## Abstract

Dust associated with various stellar sources in galaxies at all cosmic epochs remains a controversial topic, particularly whether supernovae play an important role in dust production. We report evidence of dust formation in the cold, dense shell behind the ejecta–circumstellar medium (CSM) interaction in the Type Ia-CSM supernova (SN) 2018evt three years after the explosion, characterized by a rise in mid-infrared emission accompanied by an accelerated decline in the optical radiation of the SN. Such a dust-formation picture is also corroborated by the concurrent evolution of the profiles of the Hα emission line. Our model suggests enhanced CSM dust concentration at increasing distances from the SN as compared to what can be expected from the density profile of the mass loss from a steady stellar wind. By the time of the last mid-infrared observations at day +1,041, a total amount of 1.2 ± 0.2 × 10^−2^ *M*_⊙_ of new dust has been formed by SN 2018evt, making SN 2018evt one of the most prolific dust factories among supernovae with evidence of dust formation. The unprecedented witness of the intense production procedure of dust may shed light on the perceptions of dust formation in cosmic history.

## Abstract (web summary)

By day 1,041 after explosion, SN Ia-CSM 2018evt had produced an estimated 0.01 solar masses of dust in the cold, dense shell behind the supernova ejecta–circumstellar medium interaction, ranking it as one of the most prolific dust-producing supernovae ever recorded.

## Main

The content and species of dust grains that are associated with stellar sources in galaxies at all cosmic epochs remain a controversial topic, particularly whether supernovae play an important role in dust production. Moreover, they may even carve dust-hostile environments^1,2^, considering ambient grains in any outflow of stellar wind of the supernova (SN) progenitor may become immediately sublimated and destroyed by the energetic radiation pulse produced by the SN explosion^3,4^. To date, freshly formed dust has been observed in a handful of core-collapse (CC) supernovae, both in the ejecta in situ^5–8^ and its interaction zone with the circumstellar medium (CSM) (for example, refs. ^5,9,10^). No clear observational evidence thus far shows any major formation process of dust grains in the thermonuclear runaway of ~1 *M*_⊙_ carbon/oxygen white dwarfs (WDs)^11,12^.

Type Ia supernovae are generally thought to result from thermonuclear explosions of WDs in binary systems. A rare subclass of Ia supernovae is denoted SN Ia-CSM, which is thought to be an exploding WD surrounded by a substantial amount of CSM^13^. The spectra of such events near peak luminosity are characterized by narrow Balmer emission lines superimposed together with relatively shallow Fe-group and intermediate-mass elements. SN 2002ic was the first reported case of a Ia-CSM SN that revealed large amounts of CSM seen as a strong hydrogen emission^14–17^. A number of additional Ia-CSM supernovae have been discovered and studied in detail in recent years, which include supernovae 2005gj, PTF11kx, 2012ca, 2013dn and 2015cp and a recent sample of Zwicky Transient Facility supernovae (ref. ^18^ and references therein).

**Extended Data Fig. 1:**
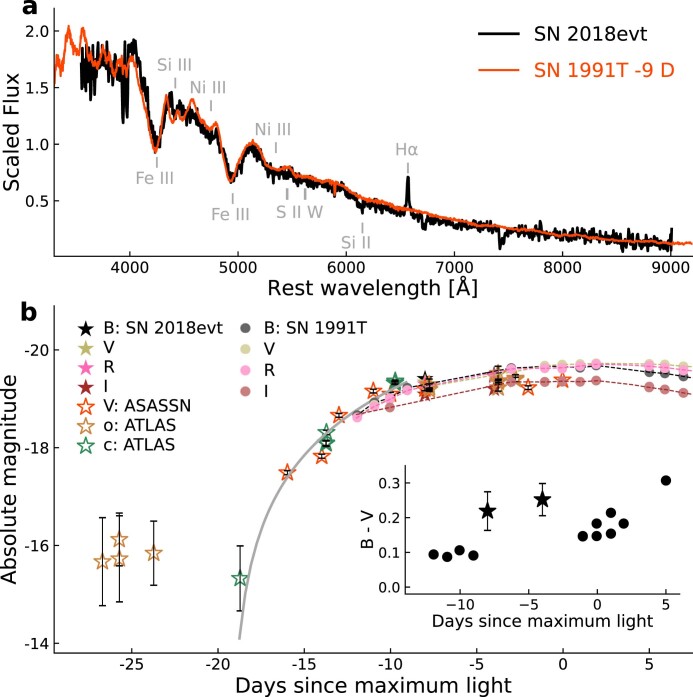
The early-time comparisons of SNe 2018evt, and 1991T. The spectrum of SN 2018evt (black curve) closely resembles the spectrum of SN 1991T at day -9 (red curve) in panel (a)^95,96^, which exhibits the strong Fe III *λ* 4404, *λ* 5129 absorptions and H*α* emission, visible Si III *λ* 4564 absorption, Ni III blends around 4750Å, 5350Å, and weak S II W and Si II *λ* 6355 as marked (for example,^98^). Panel (b) compares the early-time photometry of SN 2018evt (stars), and SN 1991T (solid circles)^97^. A power law f ∝ ${\left(\tau +{{{{\rm{t}}}}}_{{{{\rm{r}}}}}\right)}^{{{{\rm{n}}}}}$ is applied to fit the early *V* - and *c* − *b* and photometry, where *τ* = (t-${{{{{\rm{t}}}}}_{B}}^{\max }$)/(s(1+z))^23^,${{{{{\rm{t}}}}}_{B}}^{\max }$= 58352, s = 1.0 for stretch value and z = 0.02523 for the redshift of SN 2018evt. The fitting yields a rise time t_r_ = 18.76 ± 0.24 days, and a power-law index n = 1.57 ± 0.07 (grey curve). The estimated t_r_ is consistent with the *V* -b and rise time t_r_(*V*) = 20.00 ± 0.68 days of SN 1991T/1999aa-like events^23^. The interpolations of *BVRI* light curves of SN 1991T are shown in dashed curves. The inset in panel (b) compares the B -V color curves between SNe 2018evt and 1991T, indicating a color difference < ~ 0.1 mag at similar phases. The corresponding Milkyway extinction is 0.05 mag^88^ for SN 2018evt. The error bars shown represent 1 − *σ* uncertainties of magnitudes, and colors.

**Extended Data Fig. 2:**
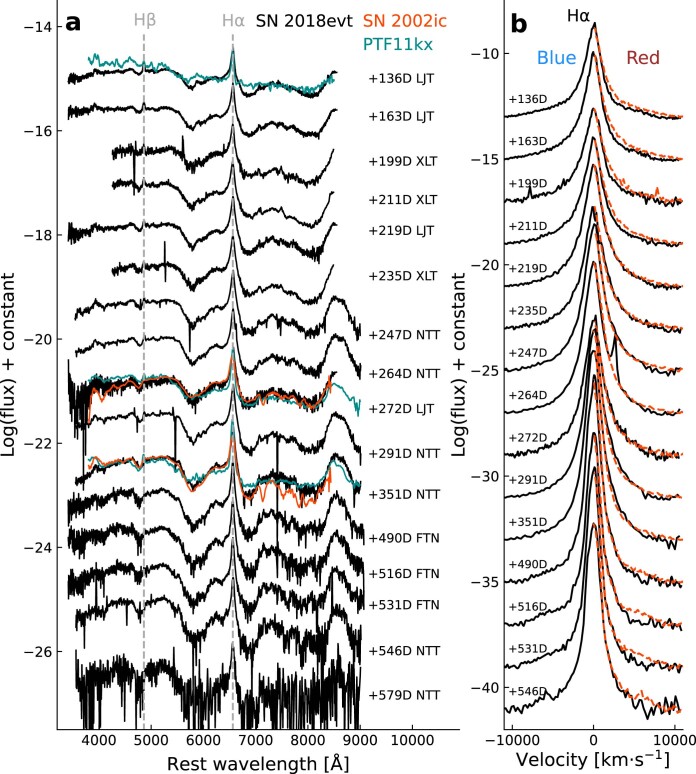
Optical spectra of SN 2018evt. Panel (a) shows optical spectra of SN 2018evt spanning from days +136 to +579 relative to *B*-band maximum. Phases and facilities are marked on the right. Spectra of SN 2018evt obtained at days +136, +272, and +351 are also compared to that of other Type Ia-CSM SNe (PTF11kx^128,129^ and SN 2002ic^14^) at similar phases. Panel (b) portrays the H*α* profile of SN 2018evt from panel (a). For each epoch, the red dashed line mirrors the spectral profile of the blue side across the peak flux of the intermediate H*α* (for example, see Extended Data Fig. 6 for two Gaussian fits to H*α*). Its deviation from the red emission wing illustrates the time-variant asymmetry of the H*α* profile.

**Extended Data Fig. 3:**
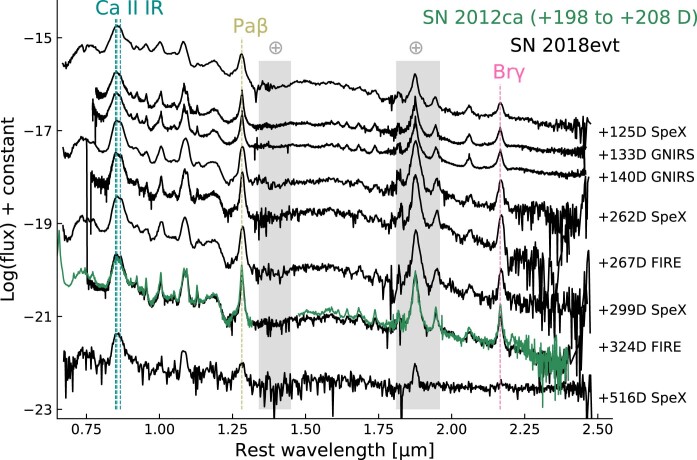
NIR spectra of SN 2018evt. Phases and facilities are marked on the right spanning from ~ +125 to +516 days relative to the B-band maximum. Several most prominent lines are labeled. The near-IR spectrum of SN 2012ca obtained at days +198 - +208 is shown for comparison^70^.

SN 2018evt (ASASSN-18ro (ref. ^19^)) is a Ia-CSM SN found in the spiral galaxy MCG-01-35-011 at redshift *z* = 0.02523 (ref. ^20^). SN 2018evt shares some common optical spectral features with typical Type Ia SN 1991T-like supernovae, as shown in Extended Data Fig. 1a. They are characterized by strong Fe iii*λ*4404 and *λ*5129 absorptions, visible Si iii *λ*4564, weak S ii W and Si ii *λ*6355 and lacking absorption features of Ca ii H and K and Ca ii infrared (IR) triplet before maximum optical light^21,22^. The early phase light curves of SN 2018evt are comparable to those of SN 1991T, as shown in Extended Data Fig. 1b. The power-law fit of the earliest light curve of SN 2018evt (≲−10 days) suggests a rise time *t*_r_ = 18.76 ± 0.24 days, which is consistent with that of SN 1991T/1999aa-like events^23^. The inset of Extended Data Fig. 1b shows the early phase *B* − *V* colour curve, which is also in general agreement with that of SN 1991T after correcting the host reddening of *E*(*B* − *V*) < 0.32 mag, which has been estimated from the equivalent width (EW) of the Na i D lines^20^. The presence of the Hα line makes it a Ia-CSM SN similar to SN 2002ic. The entire spectral sequence of SN 2018evt directly resembles other well-observed Ia-CSM supernovae events such as PTF11kx and SN 2002ic (Extended Data Fig. 2). The near-infrared (NIR) spectrum of SN 2018evt at ~324 days after the maximum is similar to that of another Ia-CSM SN candidate SN 2012ca with data at a comparable epoch (Extended Data Fig. 3).

### Results

#### Observations

**Fig. 1:**
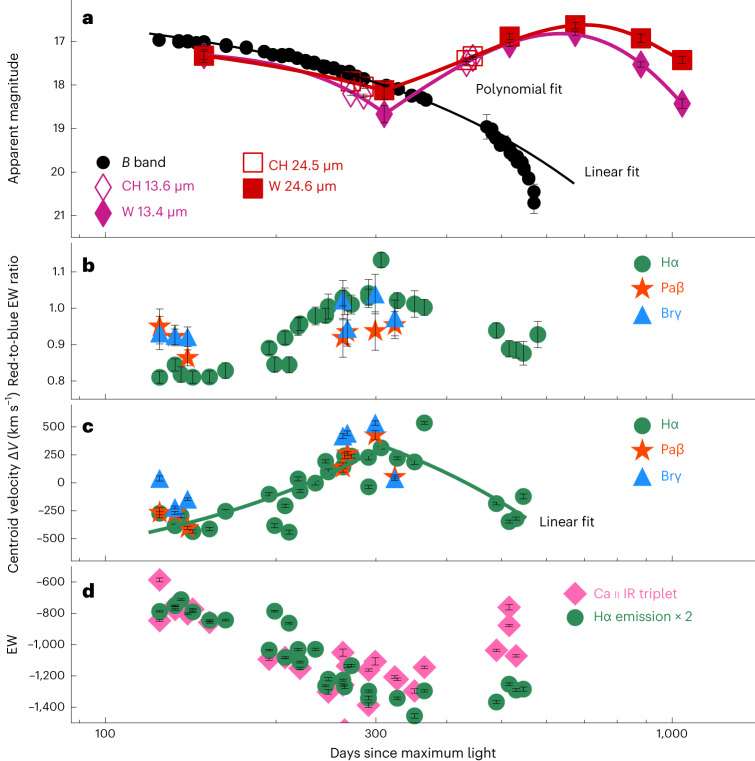
Evidence of the presence of dust in SN 2018evt. **a**, MIR and *B-*band light curves (black dots) of SN 2018evt. All phases are given relative to the estimated *B*-band maximum at MJD 58352. The Spitzer and NEOWISE observations are shown by purple diamonds and red squares as labelled. The purple and red curves fit the MIR band 1 and band 2 photometry before and after day +310, separately. The black line fits the linearly fading *B-*band photometry before day ~400, with a decline rate of 0.624 ± 0.006 mag 100 day^−1^. **b**, Red-to-blue EW ratios of Hα (green circles), Paβ (red stars) and Brγ (blue triangles) lines. **c**, Evolution of the flux-weighted centroid velocities Δ*V* of Hα, Paβ and Brγ lines labelled with the same symbols as **b**. The Δ*V* of Hα measured before and after day +310 are fitted with separate linear functions as displayed by the two green line segments. **d**, Evolution of the EW of the Ca ii IR triplet (Extended Data Table 2) and Hα lines. For the purpose of the presentation, the EW of Hα has been multiplied by a factor of 2. The error bars shown represent 1*σ* uncertainties of magnitudes, EW ratio, centroid velocity and EW. Source data

**Extended Data Fig. 4:**
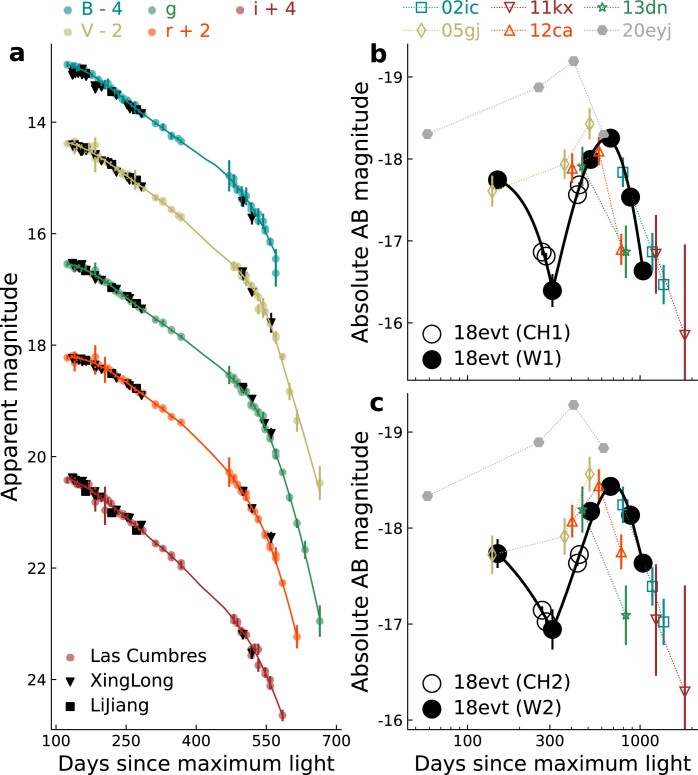
The optical and MIR light curves of SN 2018evt. Panel (a) The BV gri band light curves of SN 2018evt. Panels (b) and (c) present the ~ 3.5 *μ*m (Spitzer *CH1* and NEOWISE *W 1*) and ~ 4.6 *μ*m (Spitzer *CH2* and NEOWISE *W 2*) photometry of SN 2018evt, respectively. Black-solid lines show polynomial fits to the light curves before and after day +310. The MIR light curves of several other SNe Ia-CSM at similar phases are shown for comparison, including SNe 2002ic, 2005gj^35^, PTF11kx^69^, 2012ca, 2013dn^31,71^, and 2020eyj^72^. The error bars shown represent 1 - *σ* uncertainties of magnitudes. Source data

**Extended Data Fig. 5:**
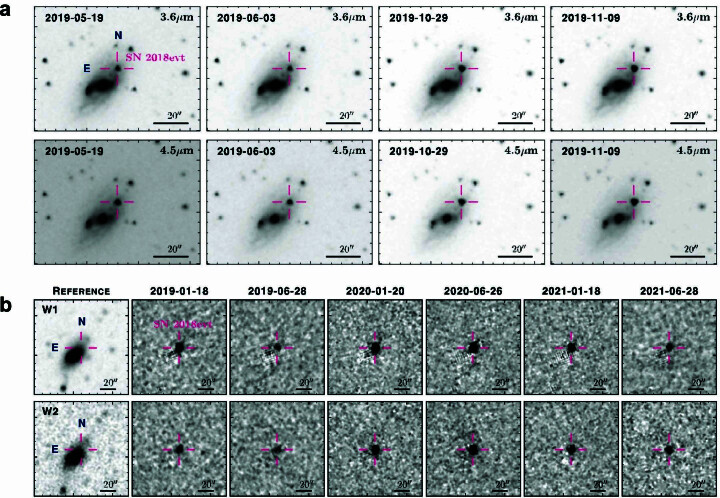
The images of SN 2018evt observed with Spitzer and NEOWISE. The first and the second rows display the Spitzer *CH1* (3.6 *μ*m) and *CH2* (4.5 *μ*m)-band images obtained from 2019-05-19 (day +271) to 2019-11-09 (day +445), respectively. The third and the fourth rows present the NEOWISE *W 1* (3.4 *μ*m) and *W 2* (4.6 *μ*m) observations of the SN 2018evt field, respectively. The left column shows the reference images constructed by coadding the pre-SN exposures between January 2017 and January 2018. The reference-subtracted images obtained from 2019-01-18 (day +149) to 2021-06-28 (day +1041) are shown in the remaining subpanels as labeled. Panels (**a**) and (**b**) display the Spitzer and NEOWISE images, respectively. In each subpanel, the magenta cross indicates the location of SN 2018evt. North is up, east is to the left.

SN 2018evt shows the characteristic spectral features of a Type Ia-CSM SN at early times together with Balmer emission lines (Extended Data Fig. 1) and the typical long-duration optical/IR light curves at late phases (Extended Data Fig. 4), indicating a continuous interaction between the expanding ejecta and a radially extending CSM. We observed SN 2018evt with the Spitzer^24^ InfraRed Array Camera (IRAC) at 3.6 μm (CH1) and 4.5 μm (CH2)^25^ in 2019 (Extended Data Fig. 5a and Extended Data Table 1). Meanwhile, the area of the SN location was scanned by the Near-Earth Object Wide-field Infrared Survey Explorer (NEOWISE) reactivation mission^26^ at 3.4 μm (W1) and 4.6 μm (W2) from 2019 to 2021^26^ (Extended Data Fig. 5b and Extended Data Table 1). The mid-infrared (MIR) fluxes of SN 2018evt exhibit an initial decline from +149 to +310 days relative to the estimated *B-*band maximum at modified Julian day (MJD) 58352; however, it is followed by an unprecedented rebrightening until the SN reached its peak luminosity in both the W1 and W2 bands at around day +674 (Fig. 1a). This behaviour is not only distinct from the steadily fading light curves in optical bandpasses but also has not been seen in any previous Ia-CSM supernovae in similar MIR filters (Fig. 1 and Extended Data Fig. 4b,c); it is, however, likely that this is due to the lack of adequate time coverage of the observations of the latter.

The optical spectral sequence of SN 2018evt spans days +125 to +579 and also reveals conspicuous temporal evolution of the asymmetric characteristic Hα profile. We measure the EW separately for the red and the blue wings of the Hα (shown in Extended Data Fig. 2, Extended Data Table 2 and ref. ^20^), Paβ and Brγ profiles (Extended Data Fig. 3 and Extended Data Table 2). The ratios of red-to-blue wing flux increase steadily from day +125 to ~+310 but turn over and decrease afterward (Fig. 1b), in pace with the MIR flux evolution. Meanwhile, the flux-weighted centroid velocity Δ*V* of the Hα line (see ‘Analysis of the spectroscopic behaviours of SN 2018evt’ for details) evolves steadily from the blueshifted side (−400 km s^−1^) to the redshifted side (+300 km s^−1^) before day +310 and thereafter moves gradually back to the blue side (−200 km s^−1^) (Fig. 1c). In addition, the evolution of the EW of the Ca ii NIR triplet also exhibits a fall and rise, in concert with the evolution of the MIR flux and the Hα line profile (see, for example, Fig. 1d).

#### Model

**Extended Data Fig. 6:**
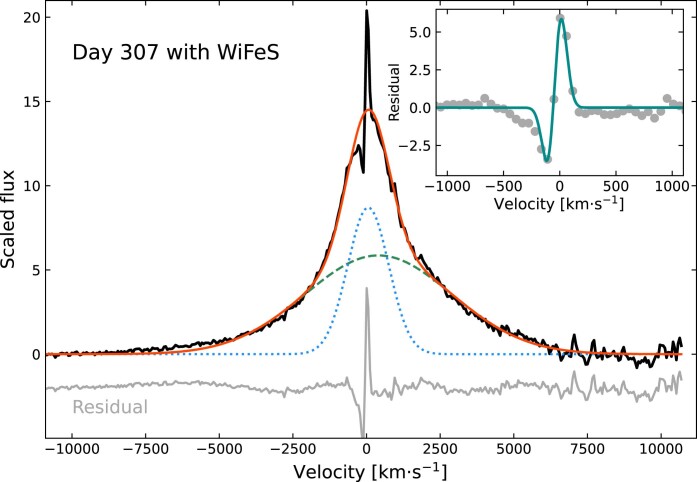
The H*α* profile of SN 2018evt observed with WiFeS at day +307 fitted with two Gaussian functions. The FWHM widths of the broad (cyan-dashed line) and intermediate (blue-dotted line) components yield 5877 ± 32 km s^−1^ and 1643 ± 12 km s^−1^, respectively. The red-solid curve gives the combination of these two components. The bottom gray line represents the H*α* profile after subtracting the broad and intermediate components. An arbitrary offset has been applied to the residual spectrum for the purpose of presentation. The inset provides a zoom-in view of the P-Cygni profile as displayed in the residual spectrum. A double-Gaussian component fit to the residual spectrum near the H*α* core is illustrated by the cyan curve. The location of the peak of the emission component suggests a redshift z =0.02561 ± 0.00019, the location of the minimum of the absorption component measures a wind velocity V_w_ = 91 ± 58 km s^−1^.

The slowly declining luminosity in the optical and NIR (Fig. 1 and Extended Data Fig. 4) and the broad, long-lived Hα line (as shown in ref. ^20^ and Extended Data Figs. 2 and 6) dominating the late-time spectra of SN 2018evt both indicate that a substantial amount of late-time emission would arise from kinetic energy from the ejecta–CSM interaction converted to radiation^27^. Such an additional energy source leads to a much slower luminosity decline (Fig. 1a) as powered by the ^56^Co → ^56^Fe decay: that is, ~0.97 mag 100 day^−1^. In such a context, a cold, dense shell (CDS) develops during the ejecta–CSM interaction, with the CDS being located at a region between the shocked CSM and the shocked ejecta^28,29^. This is the region where the SN ejecta and the CSM mix produce suitable conditions that allow the condensation of dust grains on short timescales^30–32^.

**Fig. 2:**
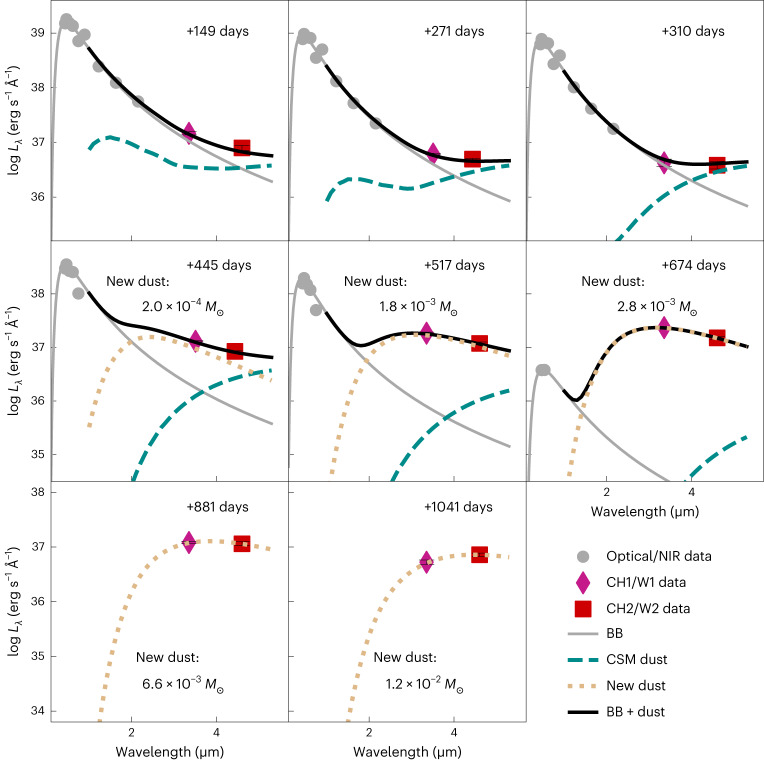
SED fitting of SN 2018evt at the rest-frame wavelength. The optical-to-NIR BVgriJHK_s_ data are fitted by a single BB before day +674. Emissions from the CSM dust calculated from our double-shell model (see ‘BB fit and dust sublimation’ and Fig. 4 for more details) are illustrated by cyan-dashed lines. Emissions from the newly formed dust are shown as yellow-dotted lines. Note that the thermal emission of the newly formed dust becomes progressively more dominant over time after day +445. The error bars shown represent 1*σ* uncertainties of the monochromatic luminosities.

**Fig. 3:**
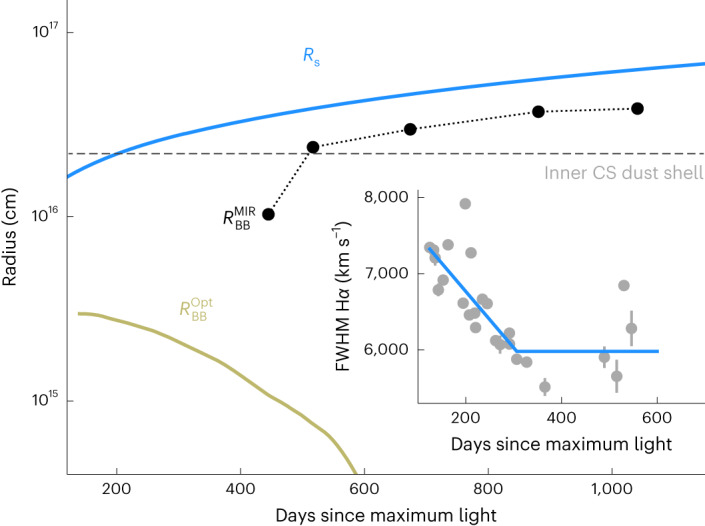
Time evolution of the different radii. The BB photospheric radius ${R}_{{{{\rm{BB}}}}}^{{{{\rm{Opt}}}}}$ and the BB dust radius ${R}_{{{{\rm{BB}}}}}^{{{{\rm{MIR}}}}}$ are derived by fitting a BB spectrum to the optical-to-NIR luminosity and the MIR flux excesses, respectively. The latter is displayed in Fig. 4, and the associated temperature of the newly formed dust can be seen from the inset of Fig. 6. The horizontal grey-dashed line indicates the inner radius of the inner shell of the CSM (2.2 × 10^16^ cm) in the double-shell model. The inset presents the temporal evolution of the FWHM width of the broad Hα line. Two blue line segments present linear fits of the data before and after day +310, respectively. The shock radius *R*_s_ was derived by equation (1) in ‘BB fit and dust sublimation’ by assuming that the shock velocity was 10,000 km s^−1^ before the first observation at day ~+120 and approximated by the FWHM width of the broad Hα afterward. The error bars shown represent 1*σ* uncertainties of FWHM.

Black-body (BB) fitting of the spectral energy distribution (SED) of SN 2018evt over the optical-to-NIR wavelength range suggests a broad temperature range of around 6,400–7,000 K during our observations. As shown in Fig. 2, the MIR flux excess is obvious and becomes progressively more dominant over time as the optical emission decreases. The MIR flux excess can be attributed to the thermal emission from dust at temperatures of 100–1,000 K (refs. ^31,33^). The photospheric radius ${R}_{{{{\rm{BB}}}}}^{{{{\rm{Opt}}}}}$ estimated from the BB fitting is shown in Fig. 3. After day +141, ${R}_{{{{\rm{BB}}}}}^{{{{\rm{Opt}}}}}$ decreases continuously with time (Fig. 3). This indicates a progressive deviation of the BB photosphere from the expanding CDS (for example, Fig. 7 of ref. ^34^), allowing the CDS to cool to a lower temperature.

**Fig. 4:**
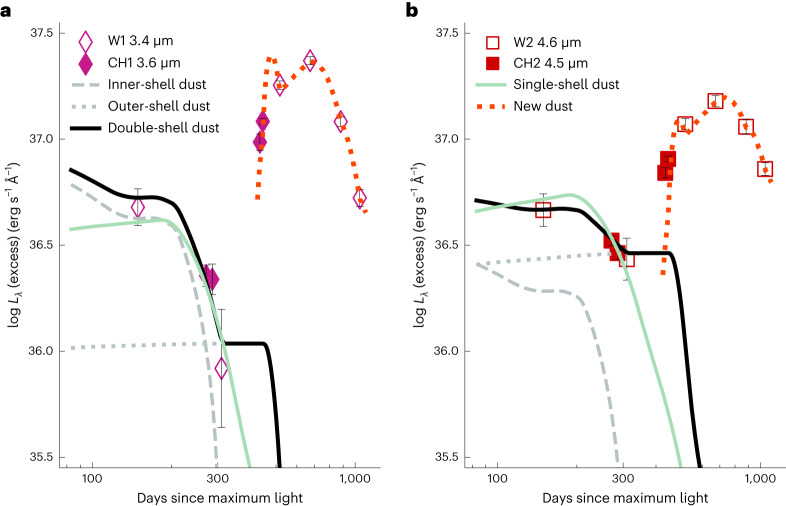
Modelling to the MIR flux excesses of SN 2018evt. The single-shell CSM dust model (solid green line) with an inner radius of 2.6 × 10^17^ cm can fit well the declining flux excess in the MIR at day ≲+310 and infer a flatter power-law index of the dust density *s* = 1.15. In the case of the steady-wind mass loss *s* = 2.0 for the double-shell model (solid black line), dust grains within the inner shell at 2.2 × 10^16^ cm were continuously destroyed by the expanding forward shock between days ~+200 and +310, causing a monotonically decreased flux excess in the MIR (dashed grey line). The presence of an outer CSM dust shell with an inner radius of 6.0 × 10^17^ cm is necessary to account for the time evolution of the flux excess before day +310 (dotted grey line). The prominent rise of the MIR flux excess of SN 2018evt after day +310, which cannot be explained by the thermal emission of any pre-existing dust content, demands a substantial amount of new dust to form promptly in the postshock regions (dotted red line). Panels (**a**) and (**b**) present the flux excesses of SN 2018evt at ~3.5 μm (Spitzer *CH1* and NEOWISE *W1*) and ~4.6 μm (Spitzer *CH2* and NEOWISE *W2*), respectively. The error bars shown represent 1*σ* uncertainties of monochromatic luminosity excesses.

We explore various radial profiles of pre-existing CSM that may account for the time-variant excess of MIR flux due to the thermal emission of dust. An initial decrease before day +310 could be attributed to a single-shell IR echo or a prominent process of dust sublimation as the forward shock runs through. The subsequent brightening after day +310 would suggest that newly formed dust accounts for the later MIR emission, in either the postshock regions of the CSM or the cooling ejecta. Assuming CSM dust density follows a power-law distribution *ρ*_dust_ ∝ *r*^−*s*^, the plausible fit to the time-variant MIR flux excesses before day +310 (Fig. 4) by searching among a grid of parameters requires a power-law index *s* = 1.15. Other free parameters include the total optical depth and the inner and outer radii of the CSM shell (‘BB fit and dust sublimation’). The shallower radial density profile (*s* = 1.15) implies enhanced dust content at larger distances from the progenitor star.

**Fig. 5:**
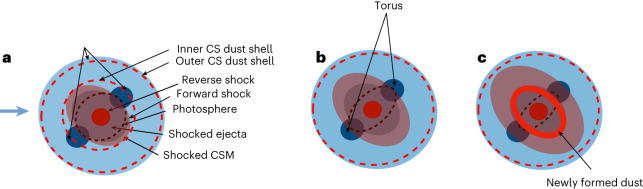
Schematic sketches of SN 2018evt at different phases. The blue arrow marks the viewing direction. The inner and outer CSM dust shells of our double-shell model are shown as dashed red circles. The double-shell CSM model to describe the SED evolution of SN 2018evt at day ≲+310 suggests inner radii of 2.2 × 10^16^ cm and 6.0 × 10^17^ cm for the inner and the outer shells, respectively. The single-shell CSM dust model infers an inner radius of 2.6 × 10^17^ cm. The sketches represent the single-shell model after deleting the inner CS dust shell in **a**. The brown-dashed ellipses approximate the location of the receding photosphere as the SN ejecta expands over time. **a**, Unshocked CSM shell and disk-like torus with pre-existing dust being destroyed by SN radiation and CSM shock. Geometric configuration before day +310 when the redshifted component of the Hα line (shown as solid blue ellipses) is blocked by the photosphere, producing blueshifted line profiles. **b**, As the SN ejecta expands and the photosphere recedes over time, more redshifted emission is revealed, resulting in a redward evolution of the line centroid as seen in Fig. 1c. **c**, When new dust forms at the postshock CDS (thick solid line), the redshifted side of Hα is blocked again, leading to blueward evolution of the line profile.

In the case of the steady dust mass loss *s* = 2, a plausible fit can also be achieved by introducing two shells of pre-existing CSM dust before day +310, namely the double-shell model. As the forward shock propagates outwards, grain sublimation takes place progressively only within the inner shell at a distance of 2.2 × 10^16^ cm, while the emitting dust grains in the outer shell, which is located at 6.0 × 10^17^ cm from the SN, remain unaffected early on (Figs. 3 and 5). Because of the lack of early time spectral coverage, we adopt an initial shock velocity *V*_s_ ≈ 10,000 km s^−1^ before day +120 based on the value estimated for SN 2002ic (refs. ^16,35^). As evidenced by the decreasing full-width at half-maximum (FWHM) width of the Hα profile (inset of Fig. 3), the forward shock expands into the inner shell of the CSM and decelerates. The progressive destruction of the inner shell dust grains leads to a continuously decreased emission in the MIR (Fig. 4). After ~+310 days, the forward shock supersedes the outer bound of the inner shell and enters a relatively low-density zone between the two CSM shells. The MIR emission becomes increasingly dominated by the relatively constant thermal emission from the outer shell. Our modelling suggests a massive outer shell of 5.2 × 10^−2^ *M*_⊙_ of dust and an inner shell of 3.2 × 10^−5^ *M*_⊙_ of dust, corresponding to two episodes of elevated dust mass loss of 2.1 × 10^−5^ *M*_⊙_ yr^−1^ and 1.8 × 10^−7^ *M*_⊙_ yr^−1^, respectively (‘BB fit and dust sublimation’).

Akin to the single-shell model, our double-shell model also suggests enhanced dust concentration at larger distances from the SN as compared to what can be expected from the density profile of the mass loss from a steady stellar wind. The dust distribution inferred from the MIR flux excesses before day +310 can be modelled in terms of a double shell, which assumes a sudden change of the density profile of the dust, or a single shell with a flatter radial profile (Fig. 4).

Both the single-shell and double-shell models are compatible with the MIR flux excesses at day ≲+310, but they cannot fit the MIR flux excesses at day >+310. After day +310, the rebrightening of SN 2018evt in the MIR demands notable contributions by additional emission sources, which can be well-attributed to the emergence of warm dust in regions behind the forward shock. As shown in Fig. 3, the BB radius ${R}_{{{{\rm{BB}}}}}^{{{{\rm{MIR}}}}}$ of the newly formed dust content fitted to the SED after day +310 increases monotonically and remains within the shock radius *R*_s_.

**Fig. 6:**
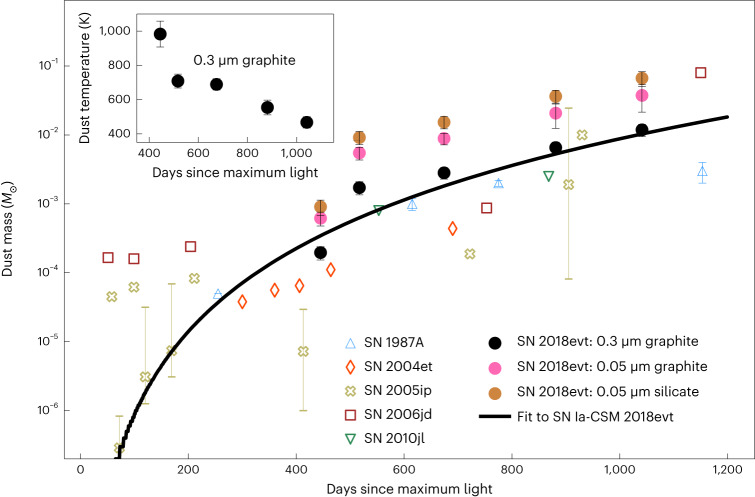
Temporal evolution of the mass of the newly formed dust. As shown by the black line, the mass of the newly formed dust of SN 2018evt can be well fitted by a power law: *M*_d_ ∝ *t*^4^ for 0.3 μm graphite grains. The dust masses calculated for graphite and silicate particles of radius 0.05 μm are also presented. The inset traces the temperature evolution of newly formed graphite dust particles of radius 0.3 μm. The dust masses estimated for Type IIP supernovae 2004et and 1987A and IIn supernovae 2005ip, 2006jd and 2010jl are also shown. The error bars shown represent 1*σ* uncertainties of masses and temperatures.

The inferred mass of the newly formed dust increases over time following a relation *M*_d_ ∝ *t*^4^ and reaches 1.2 ± 0.2 × 10^−2^ *M*_⊙_ by the time of our last observations at day +1,041 (Fig. 6 and Extended Data Table 1). The errors of the dust mass and temperatures are deduced using the Monte Carlo method via propagation of optical-to-NIR photometric errors into BB fits and the MIR photometric errors into the flux-excess calculations. The dust sublimation timescale is extremely sensitive to the temperature close to the binding energy of the dust particles^36^. Dust survival close to the shock is possible if the dust distribution is patchy or in an opaque disk, in which the self-shielding of the dust particles is important^37^. Our double-shell model assumes that a substantial amount of dust may survive the initial UV/optical emission of the SN explosion out to the inferred inner CS dust shell radius of 2.2 × 10^16^ cm, as shown in Fig. 3.

**Extended Data Fig. 7:**
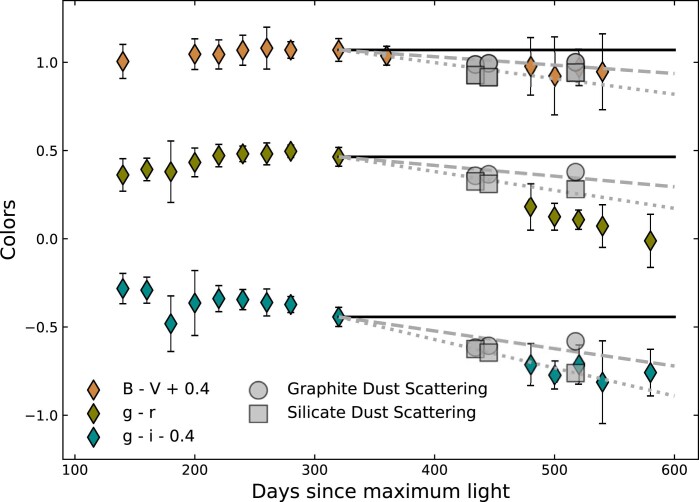
Galactic extinction-corrected B-V, g-r, and g-i color curves of SN 2018evt. All colors were binned for 20 days to increase the signal-to-noise ratio. The colors predicted by the scattering of the newly-formed dust in the post-shock CDS are also presented by gray symbols as labeled. The calculation was carried out by assuming that the intrinsic colors of SN 2018evt are identical to the values at day ~ +310, as indicated by the horizontal black line segments. The results of the 0.05 *μ*m graphite are shown by gray circles and linearly fitted by gray-dashed lines. The predicted colors of the 0.05 *μ*m silicate dust are presented by gray squares and linearly fitted by gray-dotted lines. The error bars shown represent 1 - *σ* uncertainties of colors.

Moreover, our model with dust formation is also consistent with the time evolution of the observed colours in the optical. The colours can be modelled by including the absorption and scattering effects of the newly formed dust (Extended Data Fig. 7). The increasing amount of dust after day +310 may contribute to the apparent blueward evolution of the *B*–*V*, *g*–*r* and *g*–*i* colours. An increasing amount of scattered light is expected with more dust, which leads to excess flux in the *B* and *g* bands, as shown in Extended Data Fig. 7. At even later epochs after day ~+500, the SN also exhibits accelerated fading in optical bandpasses, which is compatible with a change from the optically thin to optically thick regimes of the newly formed dust. Such a transition is similar to the dust-formation process observed in the ejecta of SN 1987A (ref. ^38^).

### Discussion

Hα emission is powered by the interaction between the ejecta and the CSM^14,15^. A thorough investigation of the time series of spectroscopy and spectropolarimetry within the first year of the SN explosion suggests that the SN ejecta expands into a dense torus of disk-like CSM^20^. Such a configuration is in good agreement with the picture depicted by the spectroscopic and MIR flux evolutions that span days +125 to +1,041. The SN ejecta running into highly asymmetric disk-like CSM leads to a high-density torus inclined at an angle towards the observer. The early blueshift of the Hα emission line is explained if the redshifted side of the shocked CSM is blocked by the photosphere, as shown schematically in Fig. 5. The redward shifts of the Hα emission line (that is, from days +125 to +310; Fig. 1) are caused by the receding photosphere as the photosphere shrinks, as proposed for the CSM configuration in PTF11iqb (ref. ^39^; their Figs. 10 and 12). After day +310, warm dust grains start to coagulate in the CDS and gradually block the receding side of the Hα line again, resulting in a blueward shift of the line profiles (Fig. 5c). After day +674, the W1–W2 colour of SN 2018evt becomes redder over time (Fig. 1a), indicating a decrease in the temperature and the MIR emission of the newly formed dust.

The presence of a highly asymmetric ejecta–CSM interaction zone is also supported by detailed spectropolarimetry of SN 2018evt, which shows a wavelength-independent degree of polarization with non-evolving position angles that is characteristic of electron/dust scattering from a highly axisymmetric configuration^15,20,40^. Despite assuming spherical symmetry, both the single-shell and double-shell distributions of the CSM dust shell provide a satisfactory description of the SED evolution of SN 2018evt spanning days +149 to +310, in particular the time-variant excess in MIR. By incorporating the geometric information obtained from spectropolarimetry^20^, the ejecta–CSM interaction process of SN 2018evt before day +310 is illustrated by the schematic sketches Fig. 5a,b. In our double-shell model, the dust in the CS wind at the radius ~2.2 × 10^16^ cm may be distributed in a disk or torus instead. The destruction and formation of the dust manifests qualitatively similar trends in the temporal evolution of the MIR excess.

The CSM masses derived from optical and optical-to-MIR luminosities in shock interaction regions are ~0.2–4.5 *M*_⊙_ (see ‘The progenitor’s mass loss’ for details; ref. ^20^), corresponding to mass-loss rates of $\dot{M}=1\times 1{0}^{-3}$ to 9 × 10^−^^2^ *M*_⊙_ yr^−^^1^. Such CSM masses estimated from the kinetic-to-radiation energy process across the shock front appear to be ~10^5^ larger compared to the amount of dust within the inner CSM shell (3.2 × 10^−5^ *M*_⊙_), which contributes most of the MIR excess before day +310 (Fig. 4). Thus a very low dust-to-gas mass ratio within the inner shell at a relatively smaller distance (2.2 × 10^16^ cm) can be inferred, which is likely caused by the prompt destruction of a substantial amount of grains in the inner shell by energetic particles from the SN^3^. On the other hand, a gas-to-dust mass ratio on the order of 100 can be inferred in the more massive (5.2 × 10^−2^ *M*_⊙_) and distant (6.0 × 10^17^ cm) outer shell, which is consistent with what is anticipated in the interstellar medium^41^. This probably means that the dust in the outer shell is much less affected by both the radiation field of the SN and the energetic particles from the shock interaction between the ejecta and the inner shell. Results similar to the outer shell can be derived by comparing the above CSM masses and the dust mass (6.0 × 10^−3^ *M*_⊙_) located at 2.6 × 10^17^ cm in the single-shell model.

The mass loss of the progenitor before the explosion is in favour of either a thermonuclear explosion from a WD + asymptotic giant branch (AGB) star system^14,42^ or a core-degenerate system in which a WD merges with the core of a massive AGB star that triggers a thermonuclear explosion at the end of a common envelope phase or shortly after^43^. The mass loss is also consistent with a WD + main sequence system for the common envelope wind model^44,45^. The progenitor systems are consistent with the measurements of the wind velocity *V*_w_ = 91 ± 58 km s^−1^ from the absorption minimum of the narrow P Cygni profiles of the Hα line (Extended Data Fig. 6 and ref. ^20^). Compared with the density profile of the dust mass loss from a steady stellar wind *s* = 2, a flatter radial profile *s* = 1.15 inferred in the single-shell model indicates enhanced dust concentration at increasing distances from the SN. The double-shell model also points to the same result that a higher dust mass-loss rate ($\dot{M}=2.1\times 1{0}^{-5}\,{{{{M}}}}_{\odot }\,{{{{\rm{yr}}}}}^{-1}$) of mass ejections is measured within the distant outer shell (6.0 × 10^17^ cm) and a lower dust mass-loss rate ($\dot{M}=1.8\times 1{0}^{-7}\,{{{{M}}}}_{\odot }\,{{{{\rm{yr}}}}}^{-1}$) is measured within the close inner shell (2.2 × 10^16^ cm). Both the single-shell and double-shell models suggest an enhanced dust presence at larger distances from the progenitor star. This shallower radial density structure results from a variable mass loss, which is likely to happen in the entire AGB evolution^46^.

The rebrightening in the MIR after day +310 can be modelled as a result of the formation of a substantial amount of warmer dust at late phases (Figs. 1, 2 and 4), distributed in a prolate shell vertical to the CSM disk (Fig. 5). It also provides a natural explanation of the red-to-blue emission-wing ratio of Hα due to uneven extinction by the newly formed dust (Fig. 1). This behaviour is also observed for the Ca ii NIR triplet, which can be similarly explained. The rapid weakening of the Ca ii NIR triplet may also indicate the depletion of calcium by dust formation. The proposed process of dust formation is corroborated by the time evolution of the EW of the Ca ii NIR triplet emission lines, which exhibits an increase after day ~+310 (see, for example, Fig. 1d). Figure 6 shows the temporal evolution of the mass of the newly formed dust of SN Ia-CSM 2018evt in our model, compared with other CC supernovae. The estimated dust mass is highly dependent on the species and size distribution of the dust grains. For graphite with a size of 0.3 μm, the dust mass grows rapidly following a power law of index 4 with the time after the explosion and reaches ~1.2 ± 0.2 × 10^−2^ *M*_⊙_ at the last epoch of the MIR observations at day +1,041 (Fig. 6). For graphite or silicate of 0.05 μm, the dust mass is about three or five times higher than the value derived for the 0.3 μm graphite dust (Extended Data Table 1), respectively. The temperature of the newly formed dust is presented in the inset of Fig. 6. A monotonically decreasing temperature from 1,000 to 500 K between day +434 and day +1,041 is likely to be mostly affected by the expansion cooling of the CDS region between the forward and reverse shocks. It may also be regulated by various heating mechanisms, including radiative heating from the SN shock, collisional heating with the ambient warm gas and energy exchange between the gas and dust^30^. As the SN ejecta expands and the dust-forming region in the CDS cools, dust grains may continue to coagulate. Depending on the duration of the timescale that the ejecta expands into the CSM, even orders of magnitude higher dust content may be produced during such a process. A notable fraction of the unburned carbon in the ejecta, if not all, can be locked in the newly formed dust. As progressively deeper layers of the ejecta move into the CDS, we may also expect a massive amount of iron and silicate dust to form in such an environment. The James Webb Space Telescope can probe the signatures of such dust in the coming years. The estimated mass (*M*_d_) and the BB emission radii ${R}_{{{{\rm{BB}}}}}^{{{{\rm{MIR}}}}}$ of the newly formed dust masses are consistent with those seen in CC supernovae at similar phases (Figs. 3 and 6 and ref. ^30^), suggesting a rapid and efficient mechanism for dust production in these supernovae.

Finally, we remark that ≲1% of CC supernovae occur in elliptical galaxies in the local universe^47^, so dust production in thermonuclear explosion supernovae Ia is a major channel of dust enrichment in early-type galaxies. Type Ia supernovae may also contribute to the dust budget in spiral galaxies^48^. SN 2002ic was the first Ia-CSM SN ever discovered and has a dwarf elliptical host^14^. The weak Hα of the host galaxy of SN 2018evt also implies overall less active star formation^49^. Even though Ia-CSM is a rare subclass of thermonuclear supernovae, the unprecedented witness of such intense production of dust grains may shed light on the perceptions of dust formation in cosmic history (‘The dust contributions of host galaxies by Ia-CSM SN events’).

## Methods

### Observations

#### Early-phase observations of SN 2018evt

The early-phase observations of SN 2018evt were conducted with the dual-channel optical/NIR camera ANDICam on the Cerro Tololo Inter-American Observatory 1.3 m telescope. Two epochs of *BVRI-*band photometry were obtained on 13 August 2018 and 17 August 2018 before the SN was too close to the Sun. ANDICam has an optical field of view (FOV) of 6.3′ × 6.3′ (0.37″ pixel^−1^) and a NIR FOV of 2.4′ × 2.4′ (0.27″ pixel^−1^). The extraction of the NIR-band photometry was not successful due to the lack of bright stars for astrometric calibration to combine the dithered images. *BVRI* point-spread function (PSF) photometry was performed on the optical images using PSFEx^50^ following the detailed prescriptions described by ref. ^51^. The PSF photometry was calibrated to the standard Pan-STARRS catalogue^52,53^ of the brightest field star at (RA, dec.) = (206.655661°, −9.680946°) (J2000) with *B* = 16.495 ± 0.034 mag, *V* = 15.812 ± 0.012 mag, *g* = 16.031 ± 0.002 mag, *r* = 15.603 ± 0.002 mag and *i* = 15.461 ± 0.003 mag. The *r-* and *i-*band photometry of this field star has been converted to the standard Johnson *RI* system^54^ following the transforming equations provided by refs. ^55–57^. The early optical light curves of SN 2018evt are shown in Extended Data Fig. 1b. We also retrieve early time photometry of SN 2018evt using the ATLAS^58,59^ forced photometry service in the *c* and *o* bands and the All-Sky Automated Survey for Supernovae (ASAS-SN^60,61^) sky patrol interface. The background flux of the ASAS-SN data of SN 2018evt has been estimated by the pre-explosion median flux recorded with the same aperture as used for the SN photometry, based on a total of 266 visits. Both ATLAS and ASAS-SN photometry are also shown in Extended Data Fig. 1b.

#### Spitzer observations

SN 2018evt was observed (primary investigator (PI): Sijie Chen) with the Spitzer^24^ IRAC at 3.6 μm (CH1) and 4.5 μm (CH2)^25^ at days +271, +286, +434 and +445. We utilized the level-2 post-basic calibrated data images from the Spitzer Heritage Archive, which were reduced by the Spitzer pipeline and resampled onto 0.6*″* pixels. Source detection and aperture photometry were performed on the images in Extended Data Fig. 5a without host subtraction using SExtractor^62^. We remark that flux difference is less than or similar to 10% for the Spitzer/IRAC photometry with and without template subtraction in ref. ^31^, which is well within the photometry uncertainty. We applied aperture corrections following the IRAC Data Handbook. The level-2 post-basic calibrated data images have been calibrated in an absolute surface-brightness unit of MJy sr^−^^1^, which can be transformed into units of uJy pixel^−^^2^ by a conversion factor of 8.4616 for the angular resolution of our IRAC images, 0.6*″* pixels. The flux was converted to absolute (AB) magnitude according to the definition *m*_AB_ = −2.5 log10(*f*) + 8.9, where *f* is in units of Jy (ref. ^63^). The AB magnitudes of SN 2018evt in the CH1 and CH2 bands are listed in Extended Data Table 1.

#### NEOWISE observations

The SN 2018evt field was also observed by the NEOWISE reactivation mission in the W1 (3.4 μm) and W2 (4.6 μm) bands since late 2013 as an extension of the WISE ALL-Sky Survey^26,64^. Using the online version of the NEOWISE Image Co-addition with Optional Resolution Enhancement (ICORE)^65,66^, we retrieve the co-added NEOWISE images that centred at SN 2018evt, with a FOV of 0.6° × 0.6° and resampled to a pixel size of 1.0*″*. Given that SN 2018evt exploded in August 2018, we take the co-added image from January 2017 to January 2018 as the reference image for background subtraction and generate the difference images for every single-visit co-added image using the Saccadic Fast Fourier Transformation (SFFT)^67^. Extended Data Fig. 5b shows the NEOWISE reference and difference images at the position centred on SN 2018evt. The time series of the differenced images clearly shows the notable variations in the brightness of SN 2018evt. The signal was notable in January 2019. After a noticeable dimming in the next six months, a dramatic rebrightening followed in 2020.

Aperture photometry was performed on the differenced images using SExtractor^62^ and calibrated to the profile-fit magnitudes in the Vega system released in the ALLWISE Source Catalogue. The photometric errors were measured on the corresponding variance images and corrected by a factor of 2.75, which gives the ratio of the input to output pixel scale (Section 13 of ref. ^66^). Such estimated photometric error *σ* for each visit is used if it is larger than the photon noise from direct photometry on the differenced images. The Vega magnitude of SN 2018evt was transformed into AB magnitude according to the magnitude offsets between the two systems^68^. The AB magnitudes of SN 2018evt in the W1 and W2 bands are listed in Extended Data Table 1. The MIR-band light curves of SN 2018evt are shown in Extended Data Fig. 4b,c, together with other Ia-CSM supernovae including 2002ic, 2005gj (ref. ^35^), PTF11kx (ref. ^69^), 2012ca (ref. ^70^), 2013dn (refs. ^31,71^), and 2020eyj (ref. ^72^).

#### Optical photometry at Las Cumbres Observatory

Extensive *BVgri* photometry spanning days +124 to +664 was obtained with the Sinistro cameras on the Las Cumbres Observatory 1 m telescope, a global network for SN observations. Images were bias subtracted and flat-field corrected using the BANZAI automatic pipeline. The background template was then subtracted from the preprocessed images, adopting the SFFT algorithm^67^. Finally, PSF photometry has been performed on differenced images using ALLFRAME^73^. We remark that the light curves of SN 2018evt before day +365 were achieved without subtracting any background template and reported in ref. ^20^. Comparisons between their direct photometry and our template-subtracted photometry obtained at similar phases suggest good agreement. In particular, the systematic magnitude differences in *BVgri* yield −0.03 ± 0.05, −0.03 ± 0.05, 0.02 ± 0.05, −0.06 ± 0.04 and −0.14 ± 0.06, respectively.

Templates for our *gri-*band exposures were directly obtained using Panoramic Survey Telescope and Rapid Response System (Pan-STARRS) cutout images for the *gri* bands. *B-* and *V-*band templates were constructed using Pan-STARRS *gr* images with the formula *B* = *g* + *w* × (*g* − *r*), *V* = *g* − *w* × (*g* − *r*). The parameter *w* was achieved by minimizing the global residual flux computed based on all field stars, which produces the cleanest subtraction. Thus the best coefficients obtained are *w* = 0.3 for *B* and *w* = 0.5 for *V*, respectively.

Zero-point calibration was conducted using local field stars by calculating the 3*σ* clipped median of the differences between instrumental magnitudes and the standard Pan-STARRS Catalog^52,53^ for *gri* and the American Association of Variable Star Observers Photometric All-Sky Survey data release 9 catalogue^74^ for *BV* (using only stars with magnitude within 10–18 mag and photometric errors *σ* < 0.1 mag). The *BVgri-*band light curves of SN 2018evt are shown in Extended Data Fig. 4a.

#### Optical photometry with XLST and LJT

Optical photometric observations of SN 2018evt were also conducted with the 60/90–cm XingLong Schmidt telescope (XLST) of the National Astronomical Observatories of China (NAOC) under a long-term Tsinghua University–NAOC Transient Survey^75^ and the Yunnan Faint Object Spectrograph and Camera (YFOSC)^76^ mounted on the 2.4–m LiJiang telescope (LJT) at the Yunnan Astronomical Observatories. SN 2018evt was observed in the imaging mode of YFOSC. Images obtained by the XLST and LJT were processed using an automatic custom pipeline based on the Image Reduction and Analysis Facility (IRAF). The pipeline reduction follows standard procedures including bias and flat-field corrections, astrometric registration, template subtraction and PSF photometry. The *BVgri* photometry is also shown in Extended Data Fig. 4a.

#### Optical spectroscopy

We also obtained 17 optical spectra of SN 2018evt. A log of the spectroscopic observations is presented in Extended Data Table 2. Six spectra were taken with the 3.6 m ESO Faint Object Spectrograph and Camera v.2 (ref. ^77^) mounted on New Technology Telescope at La Silla Observatory during the extended-Public ESO Spectroscopic Survey for Transient Objects (ePESSTO)^78^. The observations were carried out under ESO programmes 199.D-0143 (PI: Smartt) and 1103.D-0328, 106.216C (PI: Inserra). Four spectra were taken with the YFOSC/LJT in long-slit spectroscopic mode, and three were taken with the Beijing Faint Object Spectrograph and Camera^79^ mounted on the 2.16 m Xinglong telescope. One spectrum was obtained with the Wide Field Spectrograph (WiFeS) mounted on the Australian National University 2.3 m telescope at Siding Spring Observatory^80^. Three additional spectra obtained at days +490, +516 and +531 were acquired with the Folded Low Order whYte-pupil Double-dispersed Spectrograph (FLOYDS^81^) mounted on the 2.0 m telescope at Las Cumbres Observatory (Extended Data Table 2). The twin robotic FLOYDS spectrographs are mounted on the Faulkes Telescope South at Siding Spring Observatory and on the Faulkes Telescope North at Haleakala. Apart from the three late-time spectra mentioned above, another 12 spectra (Extended Data Table 2) were obtained with the same telescope from days +125 to +365 (ref. ^20^) and were also included in this paper to measure the Hα line profile (for example, red-to-blue emission-wing ratio and flux-weighted centroid velocity Δ*V*). The photometry of SN 2018evt obtained with the global network of 1 m telescopes and the 12-epoch FLOYDS spectroscopy before day +365 have been published in ref. ^20^, which focuses on the early ejecta–CSM interaction and the spectropolarimetric properties of SN 2018evt.

All optical spectra were reduced using standard IRAF routines. Flux calibration of the spectra was carried out using spectrophotometric standard stars observed at similar airmass on the same night. The spectra were further corrected for atmospheric extinction using the extinction curves of local observatories.

#### NIR spectroscopy

This Article includes eight NIR spectra (Extended Data Fig. 3 and Extended Data Table 2). Four NIR spectra were obtained with the medium-resolution 0.8–5.5 μm spectrograph and imager on the 3.0 m NASA Infrared Telescope Facility on Mauna Kea, named SpeX^82^. Two NIR spectra were acquired with the Folded port InfraRed Echellette (FIRE) spectrograph^83^ on the 6.5 m Magellan Baade telescope. Another two spectra were obtained with the Gemini NIR spectrograph (GNIRS)^84^ on the 8.2 m Gemini North telescope. The SpeX, FIRE and GNIRS spectra were reduced with Interactive Data Language codes, Spextool^85^, firehose^83^ and the XDGNIRS pipeline^86,87^, respectively.

#### Analysis of the spectroscopic behaviours of SN 2018evt

All spectra were corrected for the redshift *z* = 0.02523 of the host galaxy^20^ and extinction from the Milky Way *E*(*B* − *V*) = 0.05 mag (ref. ^88^). Three spectra lines were normalized with a pseudocontinuum by linear fitting to the spectra ranges [6250, 6350] Å and [6700, 6800] Å for Hα, [12200, 12500] Å and [13200, 13500] Å for Paβ and [21100, 21400] Å and [21900, 22200] Å for Brγ. Thus Hα, Paβ and Brγ are located at [6350, 6700] Å, [12500, 13200] Å and [21400, 21900] Å, respectively. All spectra were scaled to match the photometry in the optical bandpasses at corresponding phases and further used to measure the Hα luminosity (Extended Data Table 2) and EW (Fig. 1d). For each flux spectrum, following an approach similar to the analysis of ref. ^20^), we fit a double-component Gaussian function to the Hα profile to decompose it into a broad and an intermediate component. We found that the centre of the intermediate Gaussian component, which has a typical FWHM width of ~2,000 km s^−1^, shows only moderate shift over time until the last epoch of spectroscopy at day +579. Such behaviour is in overall good agreement with the analysis based on the spectra obtained before day +365 by ref. ^20^. The determination of the centre of the intermediate Gaussian component also allows us to compare the blue and red wings of several major emission lines. In particular, we present the red-to-blue EW ratios measured between the red and the blue wings for Hα, Paβ and Brγ features in Fig. 1b. Note that the determination of the centre of the Paβ and Brγ lines was carried out based on a single-component Gaussian fit due to the relatively low signal-to-noise ratio (S/N). The EW ratios for the Hα and Paβ lines were computed over a velocity range of −8,000 to +8,000 km s^−1^. A narrower velocity range of −3,500 to +3,500 km s^−1^ was used for the measurement of the Brγ profile.

After correcting for the redshift of the host galaxy, we define the flux-weighted centroid velocity as ${{\Delta }}V=\frac{{\lambda }_{{{{\rm{peak}}}}}-{\lambda }_{0}}{{\lambda }_{0}}\times c$, where *c* gives the speed of light and *λ*_0_ represents the rest wavelength of the line centre: that is, *λ*_0_(Hα) = 6563 Å. The flux-weighted peak wavelength, *λ*_peak_, is calculated as ${\lambda }_{{{{\rm{peak}}}}}=\frac{\int\lambda \,fd\lambda }{\int\,fd\lambda }$, where *λ* denotes the wavelength of any spectral element over the emission profile. Such a quantity weights each spectral element by its flux *f*, thus providing a more robust trace of the bulk velocity of the line-emitting zone. Figure 1c shows the Δ*V* derived for the Hα, Paβ and Brγ lines for SN 2018evt. In Fig. 1, the uncertainties of the EW ratio and centroid velocity were calculated through the Monte Carlo method, assuming that all spectra have 10% flux uncertainty.

The day +307 WiFeS^80^ spectrum obtained with a higher spectral resolution (*R* ≈ 3000) presents a well-resolved narrow Hα P Cygni profile (Extended Data Fig. 6). A two-component Gaussian fitting process suggests that the FWHM widths of the broad and intermediate components are 5,877 ± 32 km s^−1^ and 1,643 ± 12 km s^−1^, respectively. After subtracting the broad and the intermediate components from the day +307 WiFeS spectrum, we fit the residual spectrum with two separate Gaussian functions to better separate the narrow absorption and emission components of the P Cygni profile. We inferred a redshift *z* = 0.02561 ± 0.00019 by assuming the narrow-emission component peaks at the rest wavelength of Hα (inset of Extended Data Fig. 6). The wind velocity, which is measured from the blueshifted absorption minimum, gives *V*_w_ = 91 ± 58 km s^−1^. Our measurements are consistent with those reported by ref. ^20^ within the uncertainties: for example, *z* = 0.02523 ± 0.00015 and *V*_w_ = 63 ± 17 km s^−1^. The redshift values derived in both studies are also consistent with those reported to the NASA/IPAC Extragalactic Database^89,90^. Therefore, we used *z* = 0.02523 and *V*_w_ = 63 km s^−1^ (ref. ^20^) throughout the Article due to the smaller uncertainty.

### BB fit and dust sublimation

The effective BB temperatures and radii ${R}_{{{{\rm{BB}}}}}^{{{{\rm{Opt}}}}}$ were estimated by fitting a BB curve to a time series of SEDs constructed from the optical (*BVgri*) and/or NIR (*JHK*_s_) light curves of SN 2018evt. Optical photometry was obtained by the global network of the 1 m telescope at Las Cumbres Observatory, and NIR photometry was taken from ref. ^20^. The latter spans days +141 to +314 and was obtained with the Gamma-Ray burst Optical/Near-Infrared Detector^91^ mounted on the 2.2 m Max Planck Gesellschaft/European Southern Observatory telescope, operated at the La Silla Observatory in Chile. All SEDs were constructed after correcting for the *E*(*B* − *V*) = 0.05 mag Galactic extinction^88^. We adopt a distance of 103.3 Mpc for SN 2018evt following the rationale provided in ref. ^20^. Owing to the lack of early time data before day +141, we adopt the optical and NIR light curves of the well-sampled Type Ia-CSM SN 2005gj (ref. ^92^) to generate the SED of SN 2018evt during the missing phases and thereafter to calculate the MIR emission of CSM dust through the absorption and re-emission processes. Such an approximation is validated by the high similarity in prepeak and around day +140 spectra, light curve shapes and absolute brightness in optical and NIR bandpasses between supernovae 2018evt and 2005gj. In detail, supernovae 2018evt and 2005gj are both Type Ia-CSM objects (Extended Data Fig. 1 and Figure 7 in ref. ^93^). They share a similar BB temperature and radius at day ~+140 based on their multiband photometry, as shown in Fig. 3 and Table 8 of ref. ^92^. Also, the peak fluxes of SN 2005gj are comparable with those of SN 1991T (Fig. 7 of ref. ^92^), whose early phase spectrum and light curves match well with SN 2018evt (ref. ^94^), as shown in Extended Data Fig. 1 (see refs. ^95–98^ for SN 1991T).

For supernovae whose late-time emission is mostly dominated by strong ejecta–CSM interaction, their effective ${R}_{{{{\rm{BB}}}}}^{{{{\rm{Opt}}}}}$ is expected to coincide with the radius of a thin CDS^28,99^ located between the shocked CSM and the shocked ejecta. The ${R}_{{{{\rm{BB}}}}}^{{{{\rm{Opt}}}}}$ of SN 2006gy reaches its maximum value at day ~+115 (Fig. 7 of ref. ^34^), while the expansion of its CDS continues as indicated by a rather constant FWHM width of the Hα, which traces the expansion velocity of the CDS. Similar behaviour is also seen in the Type IIn SN 2006tf (Table 3 and Fig. 15 of ref. ^100^). Therefore, we suggest that the BB-fitted ${R}_{{{{\rm{BB}}}}}^{{{{\rm{Opt}}}}}$ does not trace the emitting radius of the CDS *R*_CDS_. The latter can be represented by introducing a dilution factor *ζ*, which cannot exceed unity and decreases over time^34^. The true emitting radius of the CDS is given by *R*_CDS_= ${R}_{{{{\rm{BB}}}}}^{{{{\rm{Opt}}}}}/\sqrt{\zeta }$ (refs. ^34,100^).

Figure 3 shows the ${R}_{{{{\rm{BB}}}}}^{{{{\rm{Opt}}}}}$ of SN 2018evt through BB fit to the optical-to-NIR photometry. At day +141, we measure ${R}_{{{{\rm{BB}}}}}^{{{{\rm{Opt}}}}}=3\times 1{0}^{15}$ cm. After day +141, ${R}_{{{{\rm{BB}}}}}^{{{{\rm{Opt}}}}}$ is decreasing in Fig. 3, indicating that *R*_CDS_ has departed from the corresponding BB radius at day +141 (*ζ* ≲ 1). We adopted approximately ${R}_{{{{\rm{BB}}}}}^{{{{\rm{Opt}}}}}=3\times 1{0}^{15}$ cm as the lower limit of the expanding CDS radius at day +141.

For SN ejecta whose radial density profile follows an inverse power-law distribution, *ρ*_ejecta_ ∝ *r*^−*n*^, the shock radius is given by equation (1) of ref. ^101^:

1 $${R}_{{{{\rm{s}}}}}=9.47\times 1{0}^{15}\frac{(n-2)}{(n-3)}\left(\frac{{V}_{{{{\rm{s}}}}}}{\text{3,000}{{\,{\rm{km}}}}\,{{{{\rm{s}}}}}^{-1}}\right){\left(\frac{t}{{{{\rm{years}}}}}\right)}^{(n-3)/(n-2)}\,{{{\rm{cm}}}}$$

In the literature, the shock velocity *V*_s_ is often approximated by the velocity corresponding to the FWHM width of the Hα emission line^102–104^. Assuming a typical shock velocity of 5,000–10,000 km s^−1^ (refs. ^16,105,106^), which is consistent with the Hα velocity width measured during our spectroscopic observing campaign on SN 2018evt between days +125 and +546 (inset of Fig. 3). By adopting an *n* = 8.5 ejecta density profile estimated for SN 2002ic (ref. ^17^), we estimate a forward shock radius *R*_s_ = 2.8 × 10^16^ cm at day +310.

This shock radius *R*_s_ is less than the dust evaporation radii of ~4–9 × 10^16^ cm for silicate or graphite dust^101^, assuming the SN luminosity to be *L*_bol_ = 10^43^ ergs^−1^, which falls between the maximum for SN 2018evt (10^42.8^ ergs^−1^) and the peak luminosity of SN 2005gj (10^43.7^ ergs^−1^ in Table 8 of ref. ^92^). This suggests that the pre-existing CSM dust in the single-shell model at 2.6 × 10^17^ cm and in the outer shell of our double-shell model at a distance 6.0 × 10^17^ cm are unlikely to be sublimated by the SN radiation as the dust temperature at 2.6 × 10^17^ cm can only be heated to a temperature of about 970 K, which is lower than the evaporation temperature of 1,500 K for silicate and 1,900 K for graphite^10,101,107^. The shock radius *R*_s_ is comparable to the inner radius of the inner CSM dust shell in our double-shell model (2.2 × 10^16^ cm), indicating the dust grains within the inner CSM shell are likely to be destroyed by the forward shock, if they survived the initial pulse of the electromagnetic radiation of the SN explosion due to a patchy dust distribution or in an opaque disk^37^.

#### MIR flux excess

The MIR flux excess compared to the best-fit BB SED for different epochs of observations is shown in Fig. 2. Two epochs of MIR observations were acquired with Spitzer CH1 (3.6 μm) and CH2 (4.5 μm) at days +271 and +445. The MIR observations at days +286 and +434 were not presented as they are nearly identical to the results for the day +271 and +445 observations, respectively (Extended Data Fig. 4 and Extended Data Table 1). Six more epochs of observations were acquired with NEOWISE W1 (3.4 μm) and W2 (4.6 μm) at days +149, +310, +517, +674, +881 and +1,041.

MIR emission excess typically suggests the presence of warm dust. The MIR filters used to observe SN 2018evt provide rather complete wavelength coverage of observations spanning the peak of the thermal SED from dust with temperature spanning 100 ≤ *T*_d_ ≤ 1,000 K (refs. ^33,108^). The MIR emission excess has been explained by the formation of new dust grains in a handful of CC supernovae, both in the ejecta in situ^5–8^ and in the interactions between the ejecta and the CSM (for example, refs. ^5,9,10^). Alternatively, the MIR emission excess could originate from the thermal IR emitted by dust particles that were present in a CSM before the SN event. Such primordial dust grains may have formed in the expanding matter blown from red giant stars or AGB stars^14,109^. In addition to the thermal radiation of pre-existing CSM grains, our models also include emissions from any newly formed dust to account for the extreme MIR rebrightening of SN 2018evt after day +310.

#### Modelling the emissions of SN 2018evt: a model with one or two primordial CSM shells and new dust formed in the CDS region

Dust particles in the CSM absorb some of the UV/optical photons radiated during the explosion of the SN and its ejecta interaction with the CSM and re-emit the flux in the IR bands, producing an IR echo^110–115^. Such an IR echo can be used to constrain the CSM dust properties around the SN, such as their distribution, mass and composition. IR echo models for spherically symmetric CSM shells have been developed to account for thermal emission from pre-existing CSM dust, which provides a plausible explanation for the late-time excess in the observed IR light curves of Type Ia (refs. ^115–117^) and Type II (refs. ^110,114^) supernovae. The time evolution of the IR echo is related to the ultraviolet and optical light curves of the supernovae.

At any given time, a distant observer will see the IR echo located within an ellipsoid, with the SN and the observer lying at its two foci. Such an ellipsoid traces an iso-travel-time surface of the light emitted by the SN, which expands over time. The position of any point within the ellipsoid can be expressed as (*r*, *θ*), where *r* denotes the distance from the point to the SN and *θ* represents the scattering angle. For dust particles of radius *a* located at (*r*, *θ*) and an SN located at a distance *D* from the observer, the total flux emitted by the IR echo at time *t* gives

2 $${F}_{v}(t)=\frac{{a}^{2}}{{D}^{2}}\int\nolimits_{{R}_{{{{\rm{in}}}}}}^{{R}_{{{{\rm{out}}}}}}{n}_{d}(r)\uppi {B}_{v}[{T}_{{{{\rm{d}}}}}(r,\theta ,t)]{Q}_{v}\,{d}^{3}r$$

where *R*_in_ and *R*_out_ are the radii of the inner and outer dust shell, respectively, *n*_d_ is the number density of the dust particles, and the Plank function *B*_*v*_ at frequency *ν* is determined by the dust temperature *T*_d_(*r*, *θ*, *t*), which can be estimated from the SN luminosity. *Q*_*ν*_ denotes the absorption and emission efficiency of the dust grains. The light curve of the emitting IR echo from dust distributed in a shell shows a plateau lasting for a period of 2*R*_in_/*c* (where *c* is light speed) and followed by a decline for a period of time that is related to the radial extent of the shell. Such behaviour is similar to that reported for SN 2005ip (ref. ^9^) and several other supernovae that show strong ejecta–CSM interactions (for example, refs. ^107,110,114,115^).

Assuming the CSM dust density around SN 2018evt follows an inverse power-law distribution *ρ*_dust_ ∝ *r*^−*s*^, the MIR flux emitted by the CSM dust within a single shell can be derived from equation (2). The single-shell model is assumed to be spherically symmetric and described by four parameters: the inner and outer radii of the shell (*R*_in_, *R*_out_), the optical depth in the *B* band (*τ*_B_) and the power-law index (*s*). We initially set *R*_in_ in the range of 50 ld < *R*_in_ < 150 ld (where ld is a light day) to make sure the thermal radiation of the CSM dust declines between days +100 and +300. We run the single-shell model in tens of thousands of grid points based on the four parameters (*τ*_*B*_, *R*_in_, *R*_width_, *s*) and obtain a group parameter (0.07, 100 ld, 80 ld, 1.15) to well fit the data (see the green lines in Fig. 4), where *R*_width_ = *R*_out_ − *R*_in_. A flatter radial profile of the CSM dust density was inferred from the single-shell model due to the smaller *s* compared with the value for the steady-wind mass loss of the progenitor system (*s* = 2). This suggests an increased dust concentration at increasing distances from the SN compared to what can be expected from the density profile of the mass loss from a steady stellar wind. The mass of the CSM dust within the single shell is derived to be 6.0 × 10^−3^ *M*_⊙_.

In the case of the steady-wind mass loss *s* = 2, a plausible fit can also be achieved by introducing two shells of pre-existing CSM dust before day +310. The inner shell predicted by our double-shell model was caught by the forward shock at day ~+200 (see, for example, Fig. 3). Dust grains within the inner shell are thus gradually destroyed as the shock runs. The expansion velocity of the shock was assumed to be *V*_s_ ≈ 10,000 km s^−1^ due to the lack of observations before day +120, followed by a continuous deceleration as traced by the FWHM width of the broad Hα as shown by the inset of Fig. 3. Before day +1,041, the outer shell of the CSM, which emerges at 6.0 × 10^17^ cm, remains unaffected by the forward shock. Our double-shell CSM model also provides a satisfactory fit to the monotonically decreasing MIR flux curves before day +310. The SED fits to the *BVgriJHK*_s_ and MIR-band photometry are illustrated in Fig. 2. The IR echo light curves of the two shells are shown in Fig. 4. The best-fit parameters are (*τ*_B_, *R*_in_, *R*_width_) = (0.07, 8.5 ld, 2.2 ld) for the inner shell and (0.17, 230 ld, 30 ld) for the outer shell. The total optical depth of the pre-existing dust shells in the *B* band is *τ*_B_ = 0.24, corresponding to a *V-*band extinction of *A*_V_ = 0.26 mag. We also remark that such an integrated extinction is consistent with the value estimated from the Na i D lines^20^. By assuming the CSM shells were built up by multiple epochs of pre-explosion eruptions, the derived dust mass-loss rates of the mass ejections that form the inner and outer dust shells are 1.8 × 10^−7^ *M*_⊙_ yr^−1^ and 2.1 × 10^−5^ *M*_⊙_ yr^−1^, yielding total dust masses of 3.2 × 10^−5^ *M*_⊙_ and 5.2 × 10^−2^ *M*_⊙_, respectively.

However, newly formed dust is required to explain the substantial elevation of the MIR flux excess at day >+310. The fit results of the MIR excess of SN 2018evt are also shown as dotted red curves in Fig. 4. Our fit result is achieved by assuming the newly formed dust is composed of graphite grains of radius *a* = 0.3 μm. In Fig. 6, we also present the mass of the newly formed dust as a function of time for *a* = 0.05 μm graphite and silicate grains. Dust masses estimated for other well-sampled supernovae that exhibit ejecta–CSM interactions are also presented for comparison, including the Type IIP supernovae 2004et (diamonds)^118^ and 1987A (blue upward triangles)^119–121^ and IIn supernovae 2005ip (crosses)^122,123^, 2006jd (squares)^123^ and 2010jl (green downward triangles)^10,124^. As shown in Fig. 6, the amount of dust formed by SN 2018evt is equivalent to that formed in CC supernovae.

We also remark that at day <+310, the MIR flux excess measured in band 2 (CH2 or W2) is higher than or comparable to that in band 1 (CH1 or W1), indicating a higher dust emission efficiency towards longer wavelengths. This is compatible with the large (*a* = 1.0 μm) graphite dust particles in the primordial CSM shells suggested by our single-shell and double-shell models to the time evolution of the MIR excess at day <+310.

The *a* = 0.3 μm graphite dust model provides satisfactory fits to the MIR photometry in both bands 1 and 2 at day >+310 (Figs. 2 and 4). The indicated best-fitting radius of the newly formed dust grains also falls within the 0.01–1 μm range of the typical size of the graphite dust grains (for example, refs. ^107,108^). However, the species of the newly formed dust grains may still not be inferred based on our observations as no spectral signatures of CO overtone bands at 2.3–2.5 μm were seen from our NIR spectra shown in Extended Data Fig. 3 (see also SN 2017eaw (ref. ^125^). Additionally, we are not aware of any observation of SN 2018evt conducted at 9 μm, which may discriminate the silicate and graphite dust models^108,111^. Therefore, we also present the results computed for *a* = 0.05 μm silicate and graphite dust grains in Fig. 6 and Extended Data Table 1.

### The progenitor’s mass loss

Before the SN explosion, the progenitor mass-loss rate $\dot{M}$ can be associated with the bolometric luminosity via a factor *ϵ*, which denotes the kinetic-to-radiation energy conversion efficiency. Assuming a steady stellar wind CSM (*s* = 2 in *ρ*_csm_ ∝ *r*^−*s*^ (ref. ^29^)), the bolometric luminosity *L*_bol_ can be written as

3 $${L}_{{{{\rm{bol}}}}}=\epsilon \frac{{\rm{d}}{E}_{{{{\rm{kin}}}}}}{{\rm{d}}t}=\frac{1}{2}\epsilon \frac{\dot{M}}{{V}_{{{{\rm{w}}}}}}{V}_{{{{\rm{s}}}}}^{3}$$

where *E*_kin_ represents the kinetic energy of the thin shocked shell. The efficiency factor *ϵ* is often assumed between 0.1 and 0.5 (refs. ^29,108,126,127^). We adopted *ϵ* = 0.3 and *V*_s_ = 2,000 km s^−1^, the latter being consistent with the typical FWHM velocity of the intermediate Hα component measured over our spectroscopic campaign on SN 2018evt. The wind velocity blown from the progenitor was taken from the P Cygni feature reported in ref. ^20^, *V*_w_ = 63 km s^−1^. A similar velocity was observed only in the unshocked CSM of PTF11kx (ref. ^128^) (*V*_w_ ≈ 65 km s^−1^), an 1999aa-like SN, which exhibits multiple CSM components but displays no signature of the ejecta–CSM interactions based on the early time observations^128,129^.

A sudden decrease in the optical light curves of SN 2018evt can be seen at day ~+530, indicating the formation of new dust grains in the CDS (for example, Fig. 1 and Extended Data Fig. 4). Following the prescription in ref. ^20^, we approximate the optical bolometric luminosity (*L*_Opt_) of SN 2018evt by integrating its SED at day +530 over the optical wavelength range 3,870–9,000 Å. The day +530 SED was obtained by warping the day +264 flux spectrum to match the *BVgri-*band photometry at day +530. Therefore, the estimated *L*_Opt_ = 5.2 × 10^41^ erg s^−1^ at day +530 yields a mass loss rate of

4 $$\dot{M}\approx 0.04\,{{{{M}}}}_{\odot }\,y{r}^{-1}\left(\frac{{L}_{{{{\rm{bol}}}}}}{5.2\times 1{0}^{41}\,{{{\rm{erg}}}}\,{{{{\rm{s}}}}}^{-1}}\right)\left(\frac{{V}_{\rm{w}}}{63\,{{{\rm{km}}}}\,{{{{\rm{s}}}}}^{-1}}\right)\left(\frac{0.3}{\epsilon }\right){\left(\frac{\text{2,000}\,{{{\rm{km}}}}\,{{{{{\rm{s}}}}}}^{-1}}{{V}_{{{{\rm{s}}}}}}\right)}^{3}$$

The mass of shocked CSM around SN 2018evt can be estimated by multiplying the mass-loss rate to the duration of the shock propagation (*t*_duration_) as approximated by the phase of the measurement *t*_duration_ = 530 days, which can be expressed as

5 $$\begin{array}{l}{M}_{{{{\rm{shockedCSM}}}}}=\frac{{V}_{{{{\rm{s}}}}}}{{V}_{{{{\rm{w}}}}}}\dot{M}\times {t}_{{{{\rm{duration}}}}}\approx 2.0\,{{{M}}}_{\odot }\left(\frac{{L}_{{{{\rm{bol}}}}}}{5.2\times 1{0}^{41}\,{{{\rm{erg}}}}\,{{{{\rm{s}}}}}^{-1}}\right)\\\qquad\qquad\qquad\left(\frac{0.3}{\epsilon }\right){\left(\frac{{\text{2,000}}\,{{{\rm{km}}}}\,{{{{\rm{s}}}}}^{-1}}{{V}_{{{{\rm{s}}}}}}\right)}^{2}\times\left(\frac{{t}_{{{{\rm{duration}}}}}}{530\,{{{\rm{days}}}}}\right)\end{array}$$

At such late phases of SN 2018evt, the dominant radiation source in the IR can be well-attributed to the thermal emission of newly formed dust (Fig. 2). Thanks to the MIR observations at day +517, a phase comparable to +530, we estimate the optical-to-MIR pseudobolometric luminosity *L*_Opt+MIR_ of SN 2018evt by integrating the SED over a wavelength range of 3,870 Å–5 μm (Fig. 2). The computed *L*_Opt+MIR_ = 1.2 × 10^42^ erg s^−1^ at day +517 indicates a mass-loss rate $\dot{M}\approx$ 0.09 *M*_⊙_ yr^−1^. Therefore, the corresponding mass of the shocked CSM can be estimated to be *M*_shocked CSM_ ≈ 4.5 *M*_⊙_.

At day +517, adopting a shock velocity *V*_s_ = 6,000 km s^−1^ estimated by the FWHM width of the broad Hα component (ref. ^20^ and Fig. 3) and an optical bolometric luminosity *L*_Opt_ = 5.2 × 10^41^ erg s^−1^, following equations (4) and (5), the corresponding mass-loss rate and the mass of the shocked CSM yield $\dot{M}\approx$ 0.001 *M*_⊙_ yr^−1^ and *M*_shocked CSM_ ≈ 0.2 *M*_⊙_, respectively. If we include the emission in the MIR by adopting *L*_Opt+MIR_ = 1.2 × 10^42^ erg s^−1^, the corresponding $\dot{M}$ and *M*_shocked CSM_ can be estimated to be 0.003 *M*_⊙_ yr^−1^ and 0.5 *M*_⊙_, respectively.

In the literature, the luminosity of Hα line *L*_Hα_ serves as a good indicator of the bolometric luminosity *L*_bol_ as *L*_Hα_ has found to be proportional to *L*_bol_ (refs. ^102–104,130,131^). *L*_Hα_ can be expressed as

6 $${L}_{{{{{\rm{H}\upalpha }}}}}=\frac{1}{2}{\epsilon }_{{{{\rm{{H}}}_{\upalpha }}}}\frac{\dot{M}}{{V}_{{{{\rm{w}}}}}}{V}_{{{{\rm{s}}}}}^{3}$$

where ${\epsilon }_{{{{\rm{{H}}}_{\upalpha }}}}$ denotes the efficiency of the conversion of the dissipated kinetic energy into H_α_ luminosity in the shock wave. For SN 2018evt at day ~+530, we measured *L*_Hα_ = 3.4 × 10^40^ erg s^−1^ (Extended Data Table 2). Thus we can get

7 $$\frac{{\epsilon }_{{{{\rm{{H}}}_{\upalpha }}}}}{\epsilon }=\frac{{L}_{{{{\rm{H}}}}}}{\upalpha}{{L}_{{{{\rm{Opt}}}}+{{{\rm{MIR}}}}}}\approx 0.03\times\left(\frac{{L}_{{{{\rm{{H}}}{\upalpha }}}}}{3.4\times 1{0}^{40}{{{\rm{erg}}}}\,{{{{\rm{s}}}}}^{-1}}\right){\left(\frac{{L}_{{{{\rm{Opt}}}}+{{{\rm{MIR}}}}}}{1.2\times 1{0}^{42}{{{\rm{erg}}}}{{{{\rm{s}}}}}^{-1}}\right)}^{-1}$$

We estimate *ϵ*_Hα_ ≈ 0.01 for SN 2018evt, which is comparable with the canonical value of ${\epsilon }_{{{{\rm{{H}}}_{\upalpha }}}}=0.05$ assumed in the literature^103,130^. Additionally, our computed $\frac{{L}_{{{{\rm{H\alpha }}}}}}{{L}_{{{{\rm{Opt}}}}}}\approx 0.07$ is also in general agreement with that reported in Fig. 7 of ref. ^20^.

### The dust contributions of host galaxies by Ia-CSM SN events

The far-infrared observations of the elliptical/lenticular (E/S0) galaxies by the Herschel Space Observatory suggest that the typical dust mass of such galaxies spans 10^4^–10^7^ *M*_⊙_ (refs. ^132,133^), while the average dust mass found in all types of galaxies in the local universe is 10^5.21±0.09^ *M*_⊙_. Many dwarf elliptical galaxies exhibit dust masses less than 10^5^ *M*_⊙_ (ref. ^134^).

Based on our optical-to-MIR observing campaign on SN 2018evt extended to day ~+1,000, we suggest that a total amount of ~0.01 *M*_⊙_ newly formed dust is formed in the postshocked region of the CDS. As the SN ejecta cools, more cold dust can be expected to form, as illustrated in Fig. 6. We remark that the 0.01 *M*_⊙_ new dust formed in SN 2018evt is estimated only for warm dust based on the 3.6 and 4.5 μm observations by NEOWISE and Spitzer. If we assume it estimates the typical mass of the warm dust formed in Ia supernovae, the total mass of the newly formed dust could be higher by a factor of ~10 if the bulk of the dust cools below ~30 K. Studies based on the observations of the Infrared Astronomical Satellite (IRAS) suggest ~90% of the dust in galaxies was missed by IRAS, as IRAS and Spitzer are sensitive to warm dust^135^.

Depending on the detailed physical conditions, the timescale of the grain destruction could be as long as a few Gyr, based on the revised self-consistent models of dust destruction efficiency of supernovae^136^ and other cases^137^. Moreover, the rate of Ia SN per unit mass decreases as the stellar mass of the galaxy increases (for example, Fig. 5 in ref. ^138^). In particular, Ia SN rates of 6 × 10^−13^ and 6 × 10^−14^ *M*_⊙_^−1^ year^−1^ are estimated for galaxy stellar masses of 10^9^ and 10^11^ *M*_⊙_, respectively. Assuming 0.1 *M*_⊙_ of cold dust (<30 K) is produced per Ia SN, Ia supernovae can produce on the order of 10^5^–10^6^ *M*_⊙_ of dust for typical elliptical galaxies. Given the uncertainties in dust mass in ellipticals (M_*d*_ ≤ 10^5^–10^7^ *M*_⊙_), Ia supernovae can be responsible for 10% to 100% of all the dust in elliptical galaxies. Considering that the Ia-CSM SN rate is about 0.02% to 0.2% of all Ia supernovae (ref. ^18^), the dust from Ia-CSM supernovae may be proportionally lower than the above estimate for supernovae Ia and cannot be the dominant source of dust in elliptical galaxies. We note that the effect of galaxy merging is also a dust source in E/S0 galaxies as E/S0 galaxies can capture younger galaxies together with their dust. The captured dust is usually distributed in a thin disk, but dust is also present in a diffuse environment (for example, ref. ^139^). The Ia-CSM SN contribution may also explain the diffuse dust.

### Source data

Source Data Fig. 1 *BVgri*-band photometry of SN 2018evt from 1 m network at Las Cumbres Observatory.

Source Data Extended Data Fig. 4 *BVgri*-band photometry from 2.4 m LiJiang Telescope and 60/90 cm XingLong Schmidt Telescope.

## Figures and Tables

**Extended Data Table 1:**
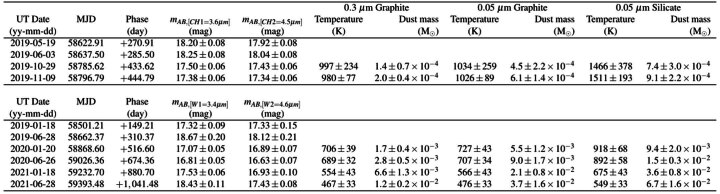
The MIR AB magnitudes of SN 2018evt, and the parameters describe the newly-formed dust

The *CH1* and *CH2* magnitudes were measured from the images taken by the Infrared Array Camera (without host subtraction) and the host-subtracted *W 1* and *W 2* magnitudes were measured from the observations by the NEOWISE reactivation mission. The presented parameters are deduced for a = 0.3 *μ*m, and a = 0.05 *μ*m graphite and silicate dust grains.

**Extended Data Table 2:**
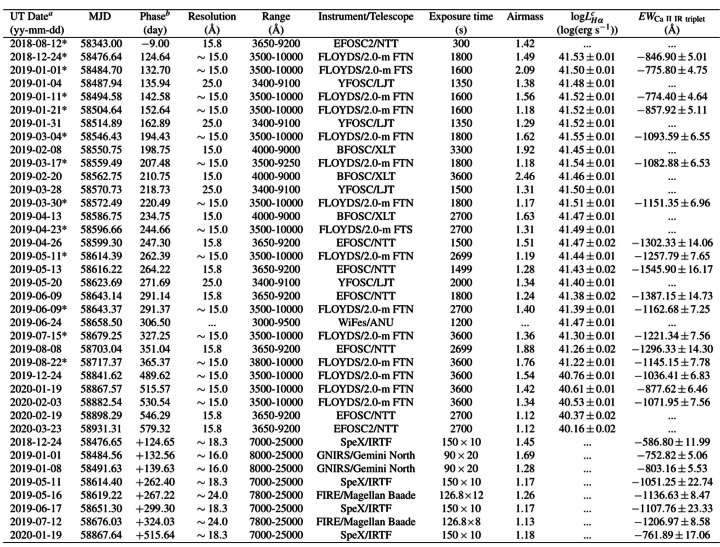
Log of the optical and NIR spectroscopic observations of SN 2018evt

The measured H*α* luminosity and the equivalent width of the Ca II NIR triplet are also listed.

a* marks the spectra that are already published in^20,94^.

b Days since B-band maximum on MJD 58352 / 2018 August 22.

c Uncertainty is derived and assumed that all spectra have 10% flux uncertainty. Note that the distance is not included. Only the Milky Way extinction is corrected with E(B-V)MW = 0.05 mag.

R_v_= 3.1.

## Data Availability

The data that support the findings of this study are openly available in the Science Data Bank at 10.57760/sciencedb.07968 (ref. ^140^) or resolve.pid21.cn/31253.11.sciencedb.07968. The global network photometry at Las Cumbres Observatory is also available in the figshare repository 10.6084/m9.figshare.21543558. All spectra will also be made publicly available via Weizmann Interactive Supernova Data Repository. ATLAS^58,59^ forced photometry service is available at fallingstar-data.com/forcedphot/queue/. The All-Sky Automated Survey for Supernovae^60,61^ sky patrol interface is available at asas-sn.osu.edu/. The Spitzer Heritage Archive is available at irsa.ipac.caltech.edu/applications/Spitzer/SHA/. The NEOWISE co-added images and ALLWISE source catalogue are available at irsa.ipac.caltech.edu/applications/ICORE/ and irsa.ipac.caltech.edu/cgi-bin/Gator/nph-scan?submit=Select&projshort=WISE. The Pan-STARRS database^52^ is available at catalogs.mast.stsci.edu/panstarrs. Source data are provided with this paper.

## Code availability

The SFFT^67^ package used for image subtraction is publicly available at github.com/thomasvrussell/sfft. The IRAF software used for spectra reduction is available at iraf.net/. The BANZAI automatic pipeline used for image reductions at Las Cumbres Observatory is available at lco.global/documentation/data/BANZAIpipeline/. The SExtractor^62^ and PSFEx^50^ software used for photometry are available at www.astromatic.net/software/sextractor/ and www.astromatic.net/software/psfex/.
